# Die Bedeutung der Kostenstruktur für die Effektivität von Staatshilfen

**DOI:** 10.1007/s10273-021-2962-x

**Published:** 2021-07-19

**Authors:** Jannis Bischof, Christopher Karlsson, Davud Rostam-Afschar, Thomas Simon

**Affiliations:** 1 LS für ABWL und Unternehmensrechnung, Universität Mannheim, Schloss Ostflügel, 68161 Mannheim, Deutschland; 2 Sonderforschungsbereichs (SFB) German Business Panel, Universität Mannheim, L9, 7, 68161 Mannheim, Deutschland; 3 LS für ABWL und Rechnungswesen, Universität Mannheim, Schloss Ostflügel, 68161 Mannheim, Deutschland

**Keywords:** H12, H81, D24

## Abstract

Die Corona-Krise führt bei vielen Unternehmen zu andauernden Umsatzeinbrüchen, die einen akuten Liquiditätsbedarf verursachen, sofern laufende Kosten nicht reduziert werden. Auf Grundlage neuer Daten des German Business Panels wird ein Klassifizierungsverfahren genutzt, das die Kostenstruktur von Unternehmen und damit die Sensitivität gegenüber Umsatzschocks sichtbar macht. Dies ist nützlich, um effektive Hilfsmaßnahmen so zu gestalten, dass sie die Beschäftigung sichern, Insolvenzen genauso wie Insolvenzverschleppung vermeiden, die speziellen Gegebenheiten von Branchen berücksichtigen und die wirtschaftliche Erholung beschleunigen.

## Abstract (en)

For many firms, the coronavirus crisis has lead to persistent sales slumps that generated urgent liquidity demand — unless fixed costs were reduced. The authors use a newly developed classification procedure that makes the cost structure of companies and, thus, their sensitivity to sales shocks visible. This is useful for designing effective relief measures that preserve corporate structures by protecting employment and avoiding insolvencies of companies with healthy prospects for a recovery during the post-crisis period. Such an approach helps to accelerate economic recovery.

Der Ausbruch der Corona-Pandemie verursachte für zahlreiche Unternehmen außergewöhnliche Umsatzeinbrüche, die seit mehr als einem Jahr andauern und angesichts der vierten Infektionswelle nur eine langsame Erholung erwarten lassen. Der Finanzminister Olaf Scholz und kürzlich Großbritanniens Schatzkanzler Rishi Sunak versprachen, jedes Mittel zu nutzen, das zur Verfügung stehe, um die negativen Folgen der Corona-Krise abzufedern (Reuters, 2021). Aber wo genau werden Hilfen am ehesten benötigt? Wie können solche Hilfen möglichst effektiv und dabei zu möglichst geringen Kosten für die Steuerzahlenden ausgestaltet werden? Wirtschaftspolitiker:innen stehen vor einem Dilemma. Einerseits kann der Einsatz von mehr Mitteln zur Unterstützung von Unternehmen die gewachsenen Unternehmensstrukturen erhalten, indem Insolvenz- und Entlassungswellen vermieden werden. Andererseits können Hilfen zu Insolvenzverschleppungen führen und durch höhere Staatsverschuldung in der Zukunft Investitionen und Wachstum mindern. Daher stellt sich die Frage, wie Unternehmenshilfen ausgestaltet sein müssen, die dem Zweck der Strukturerhaltung dienen, ohne diese Nebenwirkungen zu erzielen.

Der Beitrag zeigt, dass die Kostenstruktur für die Effektivität der Hilfen eine entscheidende Rolle spielt und identifiziert mithilfe von Daten des German Business Panels (GBP) erstmals wichtige Faktoren der internen Organisation von Unternehmen, um folgende Fragen empirisch zu quantifizieren: Welche Unternehmen trifft ein Umsatzschock härter? Gegeben welcher Kostenstruktur hat eine um einen Euro gesteigerte Staatshilfe bei gleichem Umsatzeinbruch einen stärkeren Effekt? Auf der Grundlage

**Prof. Dr. Jannis Bischof** ist Professor an der Universität Mannheim und Projektleiter im DFG-geförderten TRR 266 „Accounting for Transparency“ sowie Research Fellow am Leibniz-Institut für Finanzmarktforschung SAFE.

**Christopher Karlsson** ist wissenschaftlicher Mitarbeiter an der Universität Mannheim und im DFG-geförderten TRR 266 „Accounting for Transparency“ tätig.

**Dr. Davud Rostam-Afschar** ist akademischer Rat an den Universitäten Mannheim und Hohenheim sowie akademischer Leiter des German Business Panels im DFG-geförderten TRR 266 „Accounting for Transparency“.

**Thomas Simon** ist wissenschaftlicher Mitarbeiter an der Universität Mannheim und im DFG-geförderten TRR 266 „Accounting for Transparency“ tätig.

* Die Autoren danken der DFG für die Förderung dieses Teilprojektes im Rahmen des Sonderforschungsbereiches: Collaborative Research Center (SFB/TRR) Projektnummer 403041268 – TRR 266 Accounting for Transparency.

Abbildung 1

**Suffizienz der Coronahilfen und Überlebenserwartungen nach Fixkosten**

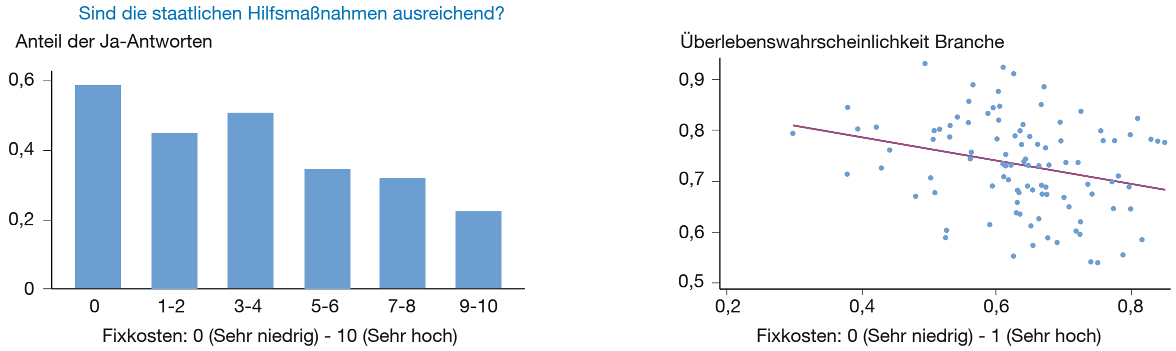

Anmerkungen: Die Fixkosten stellen hier einen betriebswirtschaftlich begründeten Indikator für die Schwierigkeit einer zeitnahen Kostensenkung dar.

Quelle: eigene Berechnungen auf Basis der Corona-Befragung des GBP (gewichtet), November bis Februar 2020/2021.

dieser empirischen Erkenntnisse wird der Vorschlag einer Strukturhilfe entwickelt, mit der Unternehmen leichter aus der Krise kommen können. Diese Strukturhilfe unterscheidet sich von bestehenden Hilfsprogrammen hinsichtlich dreier wesentlicher Aspekte:

1. Sie orientiert sich an der durchschnittlichen Umsatzentwicklung innerhalb eines Wirtschaftszweigs. Damit ist sichergestellt, dass sie nur Umsatzrückgänge berücksichtigt, die vergleichbare Unternehmen genauso betreffen, und daher plausibel durch die Pandemie begründet und nicht durch individuelle Entscheidungen oder Zufälle entstanden sind. So bleiben die Anreize bestehen, Insolvenzen möglich und der Finanzierungsbedarf kann im Vergleich zu bestehenden Programmen deutlich reduziert werden. Die Indexierung am Branchendurchschnitt der Umsatzrückgänge impliziert auch eine flexible Anpassung des Umfangs der Hilfen an die wirtschaftliche Entwicklung, sodass höhere Umsatzeinbrüche stärker gedämpft werden, die Hilfsleistungen sich aber auch selbsttätig reduzieren, wenn die Krise zurückgeht.

2. Die Strukturhilfe bezieht die Kostenstruktur eines einzelnen Unternehmens explizit durch zwei Skalierungsfaktoren ein. Zum einen berücksichtigt der Fixkostenanteil am Umsatz den Liquiditätsbedarf, zum anderen bezieht der Transmissionsfaktor, der die Auswirkungen von Umsatzschocks auf den Gewinn misst, die Verletzlichkeit von Unternehmensstrukturen mit ein.

3. Eine Orientierung am Gewinnersatz statt an den Fixkosten oder dem Umsatzrückgang bietet einen faireren und zielgerichteteren Mitteleinsatz. Die Orientierung am Gewinnersatz verbindet den Ersatz von Fixkosten mit der Idee eines fiktiven Unternehmerlohns, sodass die Strukturhilfe flexibel für Unternehmen verschiedener Art und Größe eingesetzt werden kann. Sie eignet sich damit für direkt oder indirekt durch verordnete Schließungen betroffene Unternehmen jeder Größenklasse sowie für Soloselbstständige und Freiberufler. Wie diese Ziele mit der Strukturhilfe erreicht werden können, wird anhand von Beispielen illustriert.

## Daten zur Bedeutung der Kostenstruktur in der Krise

Wieso ist die Kostenstruktur von Unternehmen entscheidend für die Erhaltung der wirtschaftlichen Struktur? Die linke Grafik in Abbildung 1 basiert auf einer Befragung von mehr als 10.000 Unternehmer:innen im Rahmen des GBP (Bischof et al., 2021a) und zeigt, dass Unternehmen mit steigender Schwierigkeit, ihre Kosten zu senken, immer seltener der Ansicht sind, dass die bisherigen Staatshilfen ausreichen. Die rechte Grafik zeigt den Zusammenhang zwischen der Schwierigkeit, Kosten zu senken, und der erwarteten Überlebenswahrscheinlichkeit auf Branchenebene. Wiederum zeigt sich, dass höhere Fixkosten mit einer geringeren Überlebenswahrscheinlichkeit zusammenhängen.

Bischof et al. (2021b) haben anhand der Daten des GBP den Einfluss der Kostenstruktur von Unternehmen auf ihre getroffenen Krisenmaßnahmen und mittelfristigen Planungsentscheidungen untersucht. Die Ergebnisse zeigen, dass Unternehmen mit hoher Kostenflexibilität um 21 % seltener steuerliche Staatshilfen in Anspruch nehmen, um 18 % seltener Investitionen verschieben und diese um 39 % seltener streichen. Außerdem entlassen sie um 29 % seltener Beschäftigte. Unternehmen mit unterschiedlicher Kostenflexibilität unterscheiden sich auch hinsichtlich ihrer mittelfristigen Pläne, da solche mit einem geringeren Anteil an Fixkosten mit höherer Wahrscheinlichkeit im nächsten Jahr investieren und mittelfristig um 7 % mehr einstellen. Die Studie nutzt einen innovativen Ansatz zur Klassifizierung der Kostenstruktur von Unternehmen: Die Idee ist, dass eine höhere Durchschlagskraft von Umsatzschocks auf die Gewinne bei ansonsten identischen Unternehmen auf Unterschiede in der Kostenstruktur schließen lässt. Sind Unternehmen mit gleichem Umsatzeinbruch überdurchschnittlich anfällig für Verluste, muss dies daran liegen, dass sie ihre Kosten nicht in entsprechendem Umfang reduzieren können. Gegeben dass der größte Anteil dieser Kosten regelmäßig zahlungswirksam ist (z. B. Miete und Lohn für Personal), hat diese Art von Unternehmen einen höheren Liquiditätsbedarf.

So wird ein Gastronomiebetrieb mit festangestelltem Personal im Vergleich zu einem Konkurrenten, der fast ausschließlich Personal auf Stundenbasis, z. B. im Rahmen von Minijobs, beschäftigt, größere Schwierigkeiten haben, die Kosten im Krisenfall zu senken. Während die Umsatzerstattung der Überbrückungshilfe im ersten Fall essenziell ist, um weiter anfallende Beschäftigtengehälter zu decken, kann der Gastronomiebetrieb im zweiten Fall seine Lohnkosten ohne vergleichbar große Hürden reduzieren und so überdurchschnittlich von den Staatshilfen profitieren. Während die grüne Kurve in Abbildung 2 die durchschnittliche Gewinnveränderung bei gegebener Umsatzveränderung für Unternehmen mit sehr starrer Kostenstruktur darlegt, zeigt die lila Kurve diesen Verlauf für Unternehmen mit einer weniger rigiden Kostenstruktur. Es wird deutlich, dass sich die Gewinne solcher Unternehmen fast proportional zu den Umsätzen verändern. Schließlich gibt es eine dritte Gruppe von Unternehmen, die ihre Kosten so flexibel anpassen können, dass die Gewinne beinahe immun gegen Umsatzschocks scheinen. Die Durchschlagkraft der Umsatzveränderung auf die Gewinne kann man anhand des Transmissionsfaktors messen:

$$\%\,\textit{Gewinnveränderung} \,/\, \%\,\textit{Umsatzveränderung} = \beta$$

Berücksichtigt die Ausgestaltung von Unternehmenshilfen diesen Faktor, können diese effektiver und zielgerichteter eingesetzt werden. Denn Unternehmen, deren Gewinne weniger empfindlich auf einen Umsatzrückgang reagieren, benötigen geringere Unterstützungsleistungen.

Das GBP liefert auch Aufschluss darüber, für welche unternehmerischen Kostenarten Staatshilfen am ehesten nötig gewesen wären. Die linke Grafik in Abbildung 3 zeigt, dass Löhne und Gehälter, der Unternehmerlohn, sowie Steuerzahlungen an erster Stelle stehen und Finanzierungslücken eine eher geringere Rolle spielen. Eine strukturerhaltende Hilfe muss also Entlassungen vorbeugen. Langfristige Personalverträge und das Know-how der Beschäftigten werden allerdings nur selten als Hürde zur Kostensenkung genannt. Die rechte Grafik zeigt, dass vor allem Planungsunsicherheit, andere langfristige Bindungen, wie laufende Mietverträge, und sonstige ge-

Abbildung 2
**Umsatz- und Gewinnveränderungen nach Kostenrigidität seit Januar 2020**

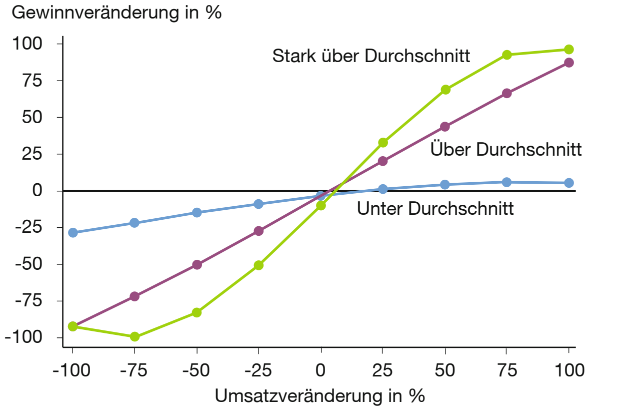

Anmerkungen: Die Kostenrigidität bestimmt den Transmissionsfaktor. Die Abbildung unterscheidet zwischen Unternehmen, deren Kostenrigidität unter, über und stark über dem Sample-Durchschnitt liegt.

Quelle: eigene Berechnungen auf Basis der Corona-Befragung des GBP, Juli bis Oktober 2020.

Abbildung 3
**Verwendung von Hilfen und Hürden der Kostensenkung**

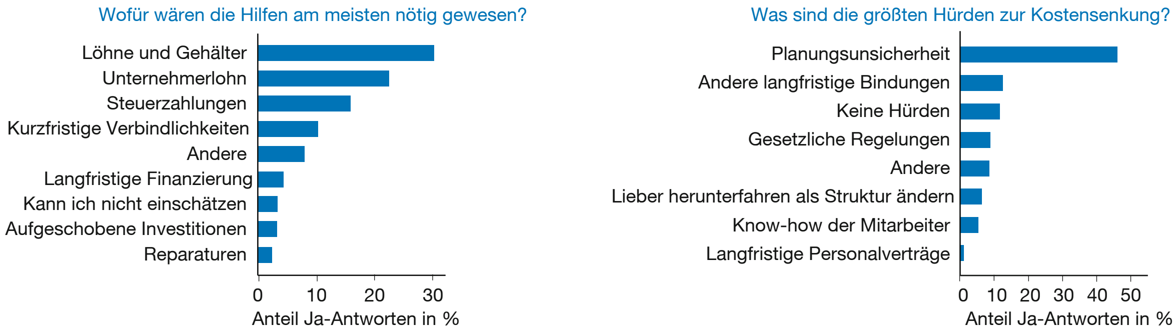

Quelle: eigene Berechnungen auf Basis der Corona Befragung des GBP, November bis Februar 2020/2021.

Tabelle 1
**Hilfsprogramme für Unternehmen 2020/2021**

| Hilfsprogramm | Formen und Maßnahmen | Explizite Berücksichtigung von Fixkosten |
|---|---|---|
| Außerordentliche Wirtschaftshilfe (November- und Dezemberhilfe) | Zuschuss pro Woche der Schließung | Pauschalisierung der Kostenzuschüsse bei 75 % der Umsatzerlöse |
| Soforthilfe Corona | Einmaliger Zuschuss nach Zahl der Beschäftigten für akute Liquiditätsengpässe | Pauschal für laufende Betriebskosten wie Mieten, Kredite für Betriebsräume, Leasingraten etc. |
| Überbrückungshilfe | Zuschuss zu den fixen Betriebskosten Überbrückungshilfe II: Sep. – Dez. 2020 Überbrückungshilfe III: Nov. 2020 – Jun. 2021 | Überbrückungshilfe II: Erstattung von<br>• 90 % Fixkosten bei Umsatzeinbruch > 70 %<br>• 60 % Fixkosten bei Umsatzeinbruch > 50 % und ≤ 70 %<br>• 40 % Fixkosten bei Umsatzeinbruch >30 % und ≤ 50 %<br>Überbrückungshilfe III (Neuerungen):<br>• 100 % Fixkostenerstattung bei Umsatzeinbruch > 70 %<br>• Breitere Definition von Fixkosten |
| Kurzarbeitergeld | Auszahlung in drei Stufen bis zu 87 % des Nettoentgelts ab dem 7. Bezugsmonat und vollständige Erstattung der Sozialversicherungsbeiträge | |
| KfW- Schnellkredit | Förderkredit mit einheitlichem Sollzins bis zu 1,8 Mio. Euro | Förderung zur expliziten Deckung von laufenden Kosten |
| Grundsicherung und Neustarthilfe | Grundsicherung für Soloselbstständige mit Corona-bedingten Umsatzeinbußen | |

Quelle: eigene Zusammenstellung.

setzliche Regelungen, wie etwa Kündigungsfristen, die Kostensenkung erschweren.

## Welche Hilfen für Unternehmen gibt es bisher?

Tabelle 1 liefert einen Überblick über die bisher verfügbaren Hilfsprogramme. Dabei sind diejenigen Maßnahmen aufgeführt, die Kostenstrukturen der betroffenen Unternehmen berücksichtigen und einen Bezug zur vorgestellten Strukturhilfe aufweisen. Die Art der Hilfen variiert in der Ausgestaltung als Kredite (KfW-Schnellkredit), als Kostenzuschüsse (November- und Dezemberhilfe, Überbrückungshilfe, Kurzarbeitergeld) oder als Grundsicherung. Obwohl der Anteil an Fixkosten eines Unternehmens eine wesentliche Determinante für dessen Krisenverhalten und Überlebenswahrscheinlichkeit darstellt, wird die Kostenstruktur bei allen verfügbaren Hilfsmaßnahmen bestenfalls eingeschränkt oder stark pauschalisierend berücksichtigt.

## Neuer Vorschlag: die Strukturhilfe

Einige bisherige Hilfsmaßnahmen wie z. B. die Überbrückungshilfe haben nach mehrfachen Überarbeitungen einen wichtigen Schritt gemacht, um die Kostenstruktur eines Unternehmens stärker einzubeziehen. Dennoch orientiert sich die Überbrückungshilfe anders als die Neustarthilfe, die sich an Soloselbstständige und Kleinstunternehmen richtet, ausschließlich an den Fixkosten. Eine solche Ausgestaltung schafft eine Reihe von Anreizen, die zu Ineffizienz führen können. Zunächst entfällt der Druck, kurzfristig laufende Kosten zu reduzieren, selbst wenn dies unter normalen Umständen geschehen wäre, und diese als Fixkosten zu behandeln, die dann zum größten Teil vom Staat erstattet werden. Umgekehrt werden Unternehmen mit sehr geringen Fixkosten, die durch flexible und zeitnahe Kostenreduktion die Krise in einem Ruhemodus überdauern können, ohne dabei Hilfsmaßnahmen in Anspruch zu nehmen, aber auch ohne weiterhin Gewinn erwirtschaften zu können, benachteiligt. Darüber hinaus führen die starr am Umsatzrückgang orientierten Anspruchsgrenzen dazu, dass schwerer betroffenen Unternehmen ein deutlich geringerer Anteil ihres – letztendlich durch staatliche Verordnungen oder Vorsichtsmaßnahmen aus gesellschaftlicher Verantwortung – entgangenen Gewinns ersetzt wird, obwohl diese einen höheren Umsatzrückgang erlitten haben. Daher haben Unternehmen bei der Beantragung von Hilfen sogar einen Anreiz, Corona-bedingte Umsatzverluste strategisch anzugeben. Abbildung 4 zeigt dies für die Überbrückungshilfe (Strichpunktlinie), die bei Umsatzeinbrüchen im Vergleich zum Vorjahresmonat zwischen 30 % und 50 % bis zu 40 % der Fixkosten ersetzt, bei Umsatzrückgängen zwischen 50 % und 70 % bis zu 60 % der Fixkosten und bei einem Umsatzrückgang von mehr als 70 % bis zu 90 % der Fixkosten erstattet.[1] Im Vergleich dazu bestehen diese Anreize bei der Strukturhilfe, der von uns vorgeschlagenen Alternative, die Schwächen bisheriger Hilfen kompensieren soll, nicht.

1 Wegen des anhaltenden Lockdowns wurde die Überbrückungshilfe III Anfang April 2021 angepasst: Nun werden bis zu 100 % der Fixkosten erstattet, sofern ein Umsatzeinbruch von mehr als 70 % erlitten wird. Obgleich die Berechnungen für diese Gruppe von Unternehmen auf einer Fixkostenerstattung von 90 % beruhen, sind die formulierten Schwächen der Überbrückungshilfe und die daraus folgenden Implikationen für die Ausgestaltung der Strukturhilfe weiterhin gültig.

Abbildung 4

**Alternative Hilfsprogramme für Unternehmen**

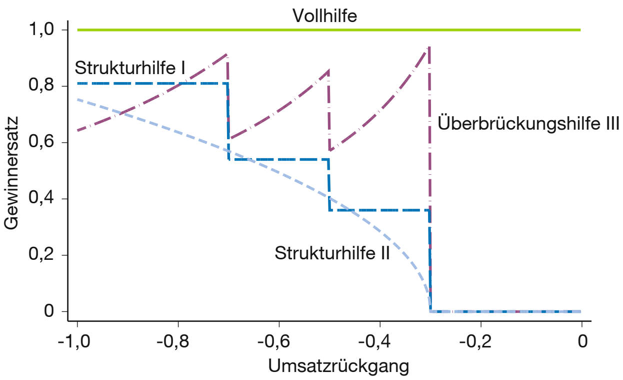

Quelle: eigene Berechnungen.

Ein weiterer Mangel bisheriger Hilfsprogramme ist, dass sie Insolvenzen quasi ausschließen, da sie nicht danach unterscheiden, welcher Teil der Umsatzverluste überhaupt durch die Pandemie verursacht wurde und welcher Teil auch unter normalen Bedingungen entstanden wäre. Außerdem sind kaum Anpassungen an branchenspezifische Gegebenheiten möglich. So könnte sich etwa die Veranstaltungs- und Kulturbranche langsamer als die Hotellerie, die Gastronomie oder die Reisebranche erholen. Zuletzt bleiben Unterschiede innerhalb der Branchen unberücksichtigt. So verzeichneten Unternehmen des stationären Einzelhandels deutlich stärkere Umsatzrückgänge im Vergleich zum Einzelhandel der Grundversorgung mit Lebensmitteln oder relativ zu Unternehmen des Versandhandels, die sogar überdurchschnittliche Gewinne verzeichnen und monopolartige Marktstellungen erreichen konnten.

## Wie funktioniert die Strukturhilfe?

Unsere Analyse der Kostenstruktur zeigt, dass Unternehmen mit starken Umsatzeinbrüchen nicht immer auch starke Verluste erleiden. Daher orientiert sich die Basisversion der Strukturhilfe am Extremfall des vollständigen Verlustersatzes, kurz Vollhilfe, die sich, wie folgt, ergibt:

$$\begin{aligned} \textit{Vollhilfe} &= \textit{Gewinn aus Vorjahresmonat - Gewinn} \\ &= \%\ \text{Umsatzveränderung} \times \text{Transmissionsfaktor } \beta \\ &\quad \times \text{Gewinn aus Vorjahresmonat.} \end{aligned}$$

Die Strukturhilfe kann auf dieser Grundlage einfach und transparent nach folgenden Kriterien berechnet werden, wobei *r* die *Umsatzveränderung* bezeichnet und $\beta$ den *Transmissionsfaktor* und die Abgrenzung der Stufen sich zur Vergleichbarkeit nach den Stufen der Überbrückungshilfe richtet:

$$\textit{Strukturhilfe I} = \begin{cases} 90\,\%\ \text{Vollhilfe},\ r < -70\,\%, \beta > 0 \\ 60\,\%\ \text{Vollhilfe},\ r \geq -70\,\%, r < -50\,\%, \beta > 0 \\ 40\,\%\ \text{Vollhilfe},\ r \geq -50\,\%, r < -30\,\%, \beta > 0 \\ 0\,\%\ \text{Vollhilfe},\ \textit{sonst} \end{cases}$$

Auf Basis dieser Berechnung werden der Liquiditätsbedarf der laufenden Kosten gedeckt, Anreize zur strategischen Angabe von Umsatzverlusten reduziert und eine gerechtere sowie zielgerichtetere Mittelzuweisung ermöglicht. Gleichzeitig wird auch der Unternehmerlohn erfasst, sodass die Hilfen als Versicherungsleistung gesehen werden können, die die Ziele der Neustarthilfe, der Überbrückungshilfe und des vereinfachten Zugangs zur Grundsicherung verbindet sowie gleichzeitig Anreize senkt, auf einen Abbau der Fixkosten bewusst zu verzichten. Dennoch bieten auch in dieser Ausgestaltung die starren Stufen bei der Abgrenzung der Umsatzrückgänge Raum für strategisches Verhalten, z. B. indem gezielt Umsatz so bemessen wird, um die nächste attraktivere Stufe zu erreichen. Eine weiter vereinfachte und transparente Version der Strukturhilfe, die Strukturhilfe II, eliminiert diese künstlichen Sprünge in den staatlichen Ersatzleistungen durch die Einführung eines Härtegradfaktors, der einen Umsatzrückgang größer als 30 % bemisst. Der Härtegrad kann z. B. wie folgt definiert werden:

$$\textit{Härtegrad} = (\textit{Umsatzveränderung} - 30\,\%)^{1/2}.$$

Die Strukturhilfe II ergibt sich dann als

$$\textit{Strukturhilfe II} = \textit{Vollhilfe} \times \textit{Härtegrad, falls } \beta > 0$$

und ist in Abbildung 4 im Vergleich zur Strukturhilfe I und der Überbrückungshilfe III illustriert.

Die weiteren Probleme bestehender Hilfsmaßnahmen können durch Erweiterungen der Strukturhilfe mit einem Skalierungsfaktor behoben werden. Bei Vollhilfe haben identische Unternehmen mit unterschiedlicher Kostenstruktur keinen Anreiz, ihr Geschäftsmodell anzupassen oder Insolvenz anzumelden. Zudem ist unklar, welcher Teil des Umsatzrückgangs auf individuelle Entscheidungen oder Zufälle zurückzuführen ist und welcher Teil der Umsatzrückgänge tatsächlich von der Pandemie verursacht wurde. Einen plausiblen Anhaltspunkt hierfür liefert die durchschnittliche Umsatzentwicklung vergleichbarer Unternehmen, z. B. der durchschnittliche Umsatzrückgang einer Branche oder, wenn Unterschiede innerhalb einer Branche zu groß sind, einer Tätigkeit, die nach weiteren für den wirtschaftlichen Einfluss der Pandemie relevanten Eigenschaften, wie dem Betrieb eines stationären Ladengeschäfts, abgegrenzt wird. Eine Skalierung mit solchen Vergleichswerten erlaubt auch bei Zahlung von Staatshilfen die Wirkung üblicher Marktkräfte, da nur Um-

Abbildung 5
**Automatische Anpassung der Hilfsleistungen an Branchenaufschwung**

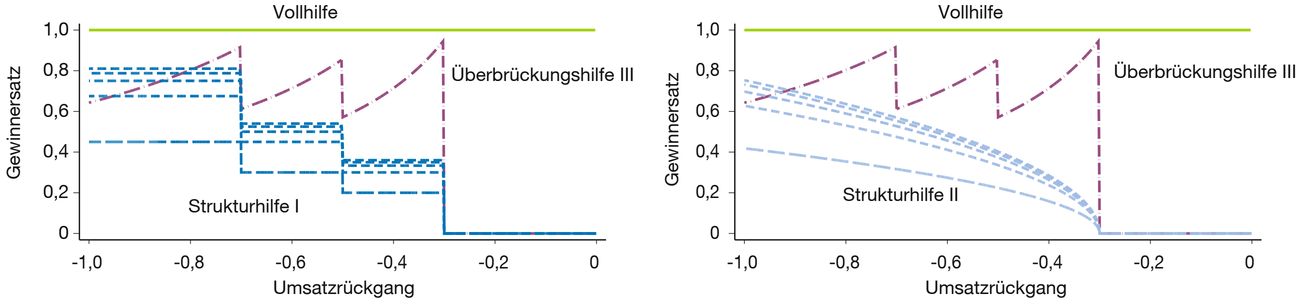

Anmerkung: Durchschnittliche Umsatzrückgänge: - 90 %, - 88 %, - 83 %, - 75 %, - 50 %.

Quelle: eigene Berechnungen.

satzeinbrüche, die Unternehmen derselben Branche im Mittel treffen, ersetzt werden.[2]

Darüber hinaus kann die Orientierung am Durchschnitt einer relevanten Vergleichsgruppe dynamisch ausgestaltet werden, sodass bei einer Verschlechterung der Wirtschaftslage ein höherer Anteil des Verlusts ersetzt wird und bei allgemeiner wirtschaftlicher Erholung die Hilfsleistungen dem niedrigeren Bedarf entsprechend sinken. Abbildung 5 illustriert die automatische Anpassung der Hilfsleistungen bei durchschnittlichen Umsatzrückgängen von - 90 %, - 88 %, - 83 %, - 75 % und - 50 %. Tabelle 2 zeigt die dynamische Anpassung anhand eines numerischen Beispiels für Umsatzrückgänge der relevanten Vergleichsgruppe von - 50 % und - 75 %.

Eine bloße Orientierung am Gewinnersatz würde jedoch genau wie eine bloße Orientierung an den Fixkosten zu kurz greifen und überhöhten Finanzierungsbedarf implizieren. Eine Skalierung der Hilfen anhand des Fixkostenanteils am gegenwärtigen Umsatz berücksichtigt dies. Das numerische Beispiel in Tabelle 2 zeigt, dass die Überbrückungshilfe III und die Strukturhilfe bei einem Fixkostenanteil von 14,3 % (10 Fixe Kosten / 70 Umsatz) identisch sind. Bei vorübergehender Schließung der Geschäftstätigkeit und vollständigem Rückgang des Umsatzes (-100 %) dämpft die Strukturhilfe den Verlust betroffener Unternehmen aber besser als die Überbrückungshilfe III.

Schließlich erlaubt die Kenntnis der Art der Kostenstruktur über die Fixkosten hinaus weitere Einsparungen von Mitteln, deren Wirkung andernfalls verpuffen würde, und eine gezielte Unterstützung von Unternehmen, die Mittel zur Strukturerhaltung benötigen. Dies kann durch die Skalierung mit dem Transmissionsfaktor erreicht werden. Da der Transmissionsfaktor nicht direkt auf das Intervall zwischen 0 % und 100 % begrenzt ist, kann eine Funktion des Transmissionsfaktors $f(\beta)$ genutzt werden. Tabelle 2 demonstriert dies für proportionale, degressive, progressive und regressive Kosten mit einer Funktion des Transmissionsfaktors, die zumindest 30 % des Corona-begründeten Verlusts ersetzt und darüber hinaus durch die Berücksichtigung der Kostenstruktur einbezieht, wie weit das individuelle Unternehmen bei gleichem Umsatzrückgang in die Verlustzone gerutscht ist.[3]

Werden vier bis auf die Kostenstruktur identische Unternehmen mit einem Umsatzeinbruch von -50 % verglichen, wird deutlich, dass die Überbrückungshilfe III diese Unterschiede in der Kostenstruktur vollständig ignoriert und daher in allen vier Beispielen zur gleichen Hilfsleistung führt. Die Strukturhilfe dagegen fällt bei degressiven Kosten höher aus, während die geringste Unterstützung bei progressiven Kosten zu verzeichnen ist. Es ist deutlich, dass die Strukturhilfe so den Ausstieg aus der Verlustzone besser als die Überbrückungshilfe erleichtert. Das Unternehmen mit degressiven Kosten, bei dem mit dem Hochfahren der Produktion oder dem Anstieg erbrachter Dienstleistungen zunächst sehr hohe Kosten anfallen, muss bei vollständigem Umsatzrückgang mit Überbrückungshilfe III zunächst einen Verlust von -47 (= -137 + 90) überwinden, bei der Strukturhilfe II aber nur einen Verlust von -20 (= -137 + 117). Ein Unternehmen mit progressiven Kosten muss bei Überbrückungshilfe III zuvor einen Verlust von nur -10 (= -100 + 90) bewältigen, bei Struktur-

2 Für Unternehmen mit Geschäftsaktivitäten in unterschiedlichen Industrien könnte der Anteil des Gesamtumsatzes in den verschiedenen Industrien ermittelt werden, um eine Orientierung dieser Umsatzanteile am zutreffenden Branchendurchschnitt zu ermöglichen.

3 Der genutzte Skalierungsfaktor ergibt sich aus $f(\beta) = log(\beta + 1) / max[log(\beta + 1), 1] + 0{,}3$.

Tabelle 2

**Die Strukturhilfe im konkreten Vergleich[1]**

| Umsatz 2019 | Umsatz 2020 | Umsatzrückgang (%) | Durchschnittlicher Umsatzrückgang (%) | Gewinn 2019 | Gewinn 2020 | Gewinnrückgang (%) | Transmissionsfaktor | Vollhilfe | Strukturhilfe I | Strukturhilfe II | Überbrückungshilfe III |
|---|---|---|---|---|---|---|---|---|---|---|---|
| Umsatzrückgang im Wirtschaftszweig -0,50 | | | | | | | | | | | |
| 140 | 105 | -25 | -50 | 40 | 5 | -88 | 3,5 | 35 | 0 | 0 | 0 |
| 140 | 70 | -50 | -50 | 40 | -30 | -175 | 3,5 | 70 | 14 | 16 | 40 |
| 140 | 35 | 75 | -50 | 40 | -65 | -263 | 3,5 | 105 | 47 | 35 | 90 |
| 140 | 0 | -100 | -50 | 40 | -100 | -350 | 3,5 | 140 | 63 | 59 | 90 |
| Umsatzrückgang im Wirtschaftszweig -0,75 | | | | | | | | | | | |
| 140 | 105 | -25 | -75 | 40 | 5 | -88 | 3,5 | 35 | 0 | 0 | 0 |
| 140 | 70 | -50 | -75 | 40 | -30 | -175 | 3,5 | 70 | 21 | 24 | 40 |
| 140 | 35 | -75 | -75 | 40 | -65 | -263 | 3,5 | 105 | 71 | 53 | 90 |
| 140 | 0 | -100 | -75 | 40 | -100 | -350 | 3,5 | 140 | 95 | 88 | 90 |
| Fixe Kosten von 10 | | | | | | | | | | | |
| 140 | 105 | -25 | -50 | 130 | 95 | -27 | 1,08 | 35 | 0 | 0 | 0 |
| 140 | 70 | -50 | -50 | 130 | 60 | -54 | 1,08 | 70 | 4 | 4 | 4 |
| 140 | 35 | -75 | -50 | 130 | 25 | -81 | 1,08 | 105 | 27 | 20 | 9 |
| 140 | 0 | -100 | -50 | 130 | -10 | -108 | 1,08 | 140 | 126 | 117 | 9 |
| Fixe Kosten von 100 | | | | | | | | | | | |
| 140 | 105 | -25 | -50 | 33 | -2 | -106 | 4,24 | 35 | 0 | 0 | 0 |
| 140 | 70 | -50 | -50 | 33 | -37 | -212 | 4,24 | 70 | 28 | 31 | 40 |
| 140 | 35 | -75 | -50 | 33 | -72 | -318 | 4,24 | 105 | 95 | 70 | 90 |
| 140 | 0 | -100 | -50 | 33 | -107 | -424 | 4,24 | 140 | 126 | 117 | 90 |
| Proportionale Kosten | | | | | | | | | | | |
| 140 | 105 | -25 | -50 | 33 | -2 | -106 | 4,24 | 35 | 0 | 0 | 0 |
| 140 | 70 | -50 | -50 | 33 | -37 | -212 | 4,24 | 70 | 19 | 21 | 40 |
| 140 | 35 | -75 | -50 | 33 | -72 | -318 | 4,24 | 105 | 63 | 47 | 90 |
| 140 | 0 | -100 | -50 | 33 | -107 | -424 | 4,24 | 140 | 84 | 78 | 90 |
| Degressive Kosten | | | | | | | | | | | |
| 140 | 105 | -25 | -50 | 3 | -32 | -1355 | 54,19 | 35 | 0 | 0 | 0 |
| 140 | 70 | -50 | -50 | 3 | -67 | -2710 | 54,19 | 70 | 28 | 31 | 40 |
| 140 | 35 | -75 | -50 | 3 | -102 | -4064 | 54,19 | 105 | 95 | 70 | 90 |
| 140 | 0 | -100 | -50 | 3 | -137 | -5419 | 54,19 | 140 | 126 | 117 | 90 |
| Progressive Kosten | | | | | | | | | | | |
| 140 | 105 | -25 | -50 | 40 | 5 | -89 | 3,54 | 35 | 0 | 0 | 0 |
| 140 | 70 | -50 | -50 | 40 | -30 | -177 | 3,54 | 70 | 18 | 20 | 40 |
| 140 | 35 | -75 | -50 | 40 | -65 | -266 | 3,54 | 105 | 59 | 44 | 90 |
| 140 | 0 | -100 | -50 | 40 | -100 | -354 | 3,54 | 140 | 79 | 74 | 90 |
| Regressive Kosten | | | | | | | | | | | |
| 140 | 105 | -25 | -50 | 33 | -2 | -107 | 4,26 | 35 | 0 | 0 | 0 |
| 140 | 70 | -50 | -50 | 33 | -37 | -213 | 4,26 | 70 | 19 | 21 | 40 |
| 140 | 35 | -75 | -50 | 33 | -72 | -320 | 4,26 | 105 | 63 | 47 | 90 |
| 140 | 0 | -100 | -50 | 33 | -107 | -426 | 4,26 | 140 | 84 | 78 | 90 |

[1] Proportionale Kosten sind berechnet mit 0,05 × Absatzmenge, degressive mit $\sqrt{10 \times \text{Absatzmenge}}$, progressive mit $(0{,}005 \times \text{Absatzmenge})^2$ und regressive mit 1.000/Absatzmenge.

Quelle: eigene Zusammenstellung und Berechnungen.

Tabelle 3
**Transmissionsfaktoren ausgewählter Industrien**

| Wirtschaftszweig | Durchschnittlicher Umsatzrückgang (%) | Durchschnittlicher Gewinnrückgang (%) | Transmissionsfaktor |
|---|---|---|---|
| Schifffahrt | -38,12 | -67,94 | 1,78 |
| Getränkeherstellung | -23,34 | -36,24 | 1,55 |
| Gastronomie | -46,44 | -52,29 | 1,13 |
| Reisebüros, Reiseveranstalter und Erbringung sonstiger Reservierungsdienstleistungen | -84,84 | -78,32 | 0,92 |
| Kreative, künstlerische und unterhaltende Tätigkeiten | -61,15 | -52,25 | 0,85 |
| Erbringung von Dienstleistungen des Sports, der Unterhaltung und der Erholung | -47,45 | -31,66 | 0,67 |

Quelle: eigene Zusammenstellung und Berechnungen.

hilfe II dagegen einen Verlust von -26 (= -100 + 74), den es leichter stemmen kann, da beim Wiederaufschwung zunächst Kosten in geringerer Höhe anfallen.

Tabelle 3 gibt einen Überblick über den Transmissionsfaktor für ausgewählte Wirtschaftszweige basierend auf den Daten des GBP. Die Klassifikation der Wirtschaftszweige basiert dabei auf dem Statistischen Bundesamt (2008).

## Fazit

Dieser Beitrag zeigt, dass die Rolle der Kostenstruktur von Unternehmen entscheidend für deren Bedarf an Liquidität während einer Krise ist. Bisherige staatliche Hilfsprogramme berücksichtigen die Kostenstruktur indes bestenfalls stark pauschalisierend. Mit dem hier vorgestellten Klassifizierungsverfahren auf Grundlage von Bilanzdaten kann gezeigt werden, welche Unternehmen von einem Umsatzschock härter betroffen sind und bei welcher Kostenstruktur die positive Wirkung von Staatshilfe, bei ansonsten identischem Umsatzeinbruch, höher ist. Auf Grundlage dieser empirischen Erkenntnisse kann eine effektivere Strukturhilfe ausgestaltet werden, die sowohl die Verletzlichkeit als auch den Liquiditätsbedarf eines Unternehmens so berücksichtigt, dass gewachsene Unternehmensstrukturen während einer Krise erhalten werden. Dies hilft gleichzeitig, Mitarbeiterentlassungen und Insolvenzen gesunder Unternehmen zu vermeiden und den Einsatz staatlicher Mittel zu reduzieren. Die einfache und transparente Umsetzung berücksichtigt den Durchschnitt der Umsatzrückgänge relevanter Vergleichsunternehmen. Dies trägt dazu bei, dass sich die Bemessung der Staatshilfen gezielter an den von der Corona-Pandemie verursachten Umsatzeinbrüchen ausrichtet und sich der Umfang der Hilfsmaßnahmen selbsttätig an den jeweils aktuellen Bedarf, abhängig von der allgemeinen wirtschaftlichen Entwicklung, anpasst. Dadurch sind auch während der Krise Insolvenzen und die Wirkung wirtschaftlicher Anreize zum effizienten Kosteneinsatz wie in normalen Zeiten möglich. Die Orientierung am Gewinnersatz statt am Umsatzrückgang oder an Fixkosten erlaubt somit einen flexibleren und gerechteren Mitteleinsatz. Dieser Beitrag fokussiert sich auf die konkrete Ausgestaltung der Ausgaben, jedoch bietet die Form der Finanzierung dieser Mittel, etwa über einen Fonds oder als Kredit anstatt über Steuergelder, weiteren Raum für wichtige Verbesserungen.
